# Mixed Epithelial and Stromal Tumor Family of Kidney (Adult Cystic Nephroma, Mixed Epithelial and Stromal Tumor): Retrospective Clinicopathological Evaluation

**DOI:** 10.5146/tjpath.2022.01575

**Published:** 2022-09-15

**Authors:** Hale Demır, Zehra Sahın, Oktay Ozman, Muhammed Demırbılek, Sami Berk Ozden, Iclal Gurses, Haydar Durak, Nesrin Uygun, Bulent Onal

**Affiliations:** Department of Pathology, Amasya University, Faculty of Medicine, Amasya, Turkey; Gaziosmanpaşa Training and Research Hospital, Istanbul, Turkey; Department of Urology, Netherlands Cancer Institute, Antoni Van Leeuwenhoek Hospital, Amsterdam, Netherlands; Istanbul University-Cerrahpasa, Cerrahpasa Faculty of Medicine, Istanbul, Turkey; Department of Pathology, Istanbul University-Cerrahpasa, Cerrahpasa Faculty of Medicine, Istanbul, Turkey; Acibadem Hospital, Istanbul, Turkey

**Keywords:** Mixed epithelial stromal tumor, Adult cystic nephroma, Kidney, Renal cyst

## Abstract

***Objective:*** Tumors belonging to the mixed epithelial stromal tumor family (MESTF) are rare; thus clinicopathological experience about them are limited. Each epithelial and stromal component shows different patterns in these tumors.

***Material and Method:*** Clinicopathological features of 11 MESTF cases that were diagnosed between 2000 and 2021 at a single center were evaluated.

***Results:*** Ten of the 11 patients were female (F:M = 10:1). The mean age of the females was 47 (31-63) years; the male patient was 45 years old. The mean tumor diameter was 6.7 (3.5-19) cm. All tumors had varying proportions of cystic and solid components. Eight cases were well circumscribed, and the others had distinct but irregular borders. Two of the tumors with irregular borders were bulging into the renal sinus. The epithelial component was dominant in most cases. In the epithelial component, macrocyst, microcyst, and tubules were the most common patterns and the most common types of lining epithelium were flat, cuboidal and hobnail. The stromal component was variable in most cases and included hypocellular (mostly collagenous) and cellular areas. In most cases, the cellular stroma had an ovarian-like appearance. Among the other features observed, hyalinization and dystrophic calcification were common. The positivity for estrogen and progesterone receptor in the stromal component was observed in almost all female cases.

***Conclusion:*** MESTF, which has distinctive features, should be considered in the differential diagnosis of cystic kidney tumors.

## INTRODUCTION

Cystic nephroma (CN) and mixed epithelial and stromal tumor (MEST) are rare complex kidney tumors consisting of epithelial and stromal elements (1–3). Adult CN (ACN) and MEST have clinic, morphologic and immunophenotypic similarities (4,5). It was also shown that ACN and MEST exhibited similar mRNA expression profiles that were distinct from the other renal tumors (5). In the 2016 World Health Organization (WHO) classification, these two entities were accepted as same type of tumors that could be more cystic (ACN) and more solid (MEST), and were grouped under the title of “mixed epithelial and stromal tumor family” (MESTF) (6,7). In contrast, pediatric CN (PCN) is accepted as a different entity. Although the role of DICER 1 mutation was well established in PCN, there is no evidence revealing the presence/absence of this mutation in ACN and MEST yet (8).

MESTF cases were reported mostly among perimenopausal women (1–3). Clinically, they most commonly present with palpable abdominal mass, flank pain, and hematuria (2,9). Radiologically, these are mostly well-circumscribed, multicystic masses with solid components (9,10). They can be localized in the medulla or cortico-medullar region or can be centered in the renal pelvis. Medullar tumors may bulge into the renal pelvis (7).

The majority of the tumors are well circumscribed, with capsules of variable thickness, containing smooth muscle (11). Each of the epithelial and stromal components may show different patterns. The epithelial component, which rarely contains complex structures, shows a tubular and cystic pattern lined with flat, cuboidal, and hobnail type epithelium (4,5). Heterologous epithelial differentiations (müllerian, urothelial, etc.) can be also seen (7). The stroma can be hypocellular or hypercellular and is often variable. The hypocellular stroma is mostly collagenous while the hypercellular stroma is mostly ovarian-type. It may contain different mesenchymal metaplasias (smooth muscle, lipomatous, ect.) (1–4). Minimal atypia can be seen in both of the components, but mitosis and necrosis are rare pathological findings (7). Immunohistochemical positivity for estrogen and progesterone receptors in the stromal component were reported in most cases (1,4).

Most cases are benign and radical/partial nephrectomy is the curative treatment option (1,12). However, recurrence and malignant transformation were reported in a limited number of cases (12–15).

The aim of this retrospective study was to evaluate the clinicopathological features of 11 MESTF cases.

## MATERIALS and METHODS

The study protocol was approved by the Clinical Research Ethics Committee of a local university (No: 57, Date: 06/05/2021).

This study included 11 cases that were diagnosed with ACN and MEST in the pathology department of an university hospital, between 2000 and 2021. For each case, clinical data including age, gender, symptoms, hormonotherapy history, radiological features, surgery type, and follow-up were recorded. In addition, by using the pathological reports, macroscopic features of the tumor such as size, borders, cystic or solid appearance, and extension beyond the parenchyma were recorded.

Hematoxylin & Eosin stained slides of each case were reevaluated. At low magnification, border characteristics of the tumors and ratio of epithelial and stromal components of each tumor were evaluated. Cysts, which are an element of the epithelial component, were classified according to their size; microcyst <5 mm, macrocyst >5 mm. Afterwards, the histopathological features of the components were examined in details and recorded. Cytological atypia, mitosis and necrosis were also noted.

Immunohistochemical studies were performed for ER and PR expression in all cases. The only tumor resected from a male patient was also examined for androgen receptor (AR) expression. The findings related to these were recorded from pathology reports.

## RESULTS

### Clinical Findings

Ten of 11 patients were female (F:M = 10:1). The mean age of the female cases was 47 (31-63) years; the male patient was 45 years old.

The complaints reported were flank pain, abdominal pain, abdominal mass, hematuria and polyuria in order of frequency. A 61-year-old female patient had hormone replacement therapy for 2 years in the postmenopausal period. There was no hormonotherapy history of other 8 cases including the male patient. This data could not be reached in 2 cases. Six (54.5%) of the tumors were localized in the left kidney and 5 (45.5%) were in the right. The tumors were localized in the middle portion of the kidney in 8 cases (72.7%) and in the polar region in the other 3 cases (27.3%). Radiologically, the tumors were defined as a complicated cystic lesion in 4 cases, a cystic-solid lesion in 2 cases including the male patient, and a solid mass in 2 cases; these data could not be reached for 3 cases. Partial nephrectomy was performed in 2 cases and radical nephrectomy in 9. The follow-up periods of 10 cases were known and ranged from 4 to 258 (mean 81) months. There was no recurrence or metastasis in any of the cases. Clinical findings were summarized in Table 1.

**Table 1:** Clinical findings of the MESTF cases.

	**Case 1**	**Case 2**	**Case 3**	**Case 4**	**Case 5**	**Case 6**	**Case 7**	**Case 8**	**Case 9**	**Case 10**	**Case 11**
**Age**	63	49	40	58	35	45	45	39	61	50	31
**Gender**	F	F	F	F	F	F	M	F	F	F	F
**Symptoms**	abdominal mass	abdominal mass	flank pain	abdominal mass	abdominal pain, hematuria	abdominal pain	polyuria	flank pain	abdominal pain	flank pain	hematuria, flank pain
**Hormone therapy history**	absent	absent	absent	unknown	absent	absent	absent	absent	postmenopausal 2 years	absent	unknown
**Radiological features**	unknown	unknown	unknown	complicated cystic lesion	complicated cystic lesion	complicated cystic lesion	lobulated cystic-solid lesion	solid lesion	cystic-solid lesion	solid lesion	complicated cystic lesion
**Surgery**	N	N	N	N	N	PN	PN	N	N	N	N
**Tumor side**	right	left	right	left	right	left	left	right	right	left	left
**Tumor localization**	middle	lower pole	unknown pole	middle	middle	middle	middle	middle	upper-middle	lower pole	lower-middle
**Follow-up**	NSD for 258 months	NSD for 148 months	NSD for 141 months	unknown	NSD for 121 months	NSD for 43 months	NSD for 54 months	NSD for 22 months	NSD for 10 months	NSD for 9 months	NSD for 4 months

**F:** Female, **M:** Male, **N:** Nephrectomy, **PN:** Partial nephrectomy, **NSD:** No signs of disease.

### Macroscopic Features

**Figure 1:**
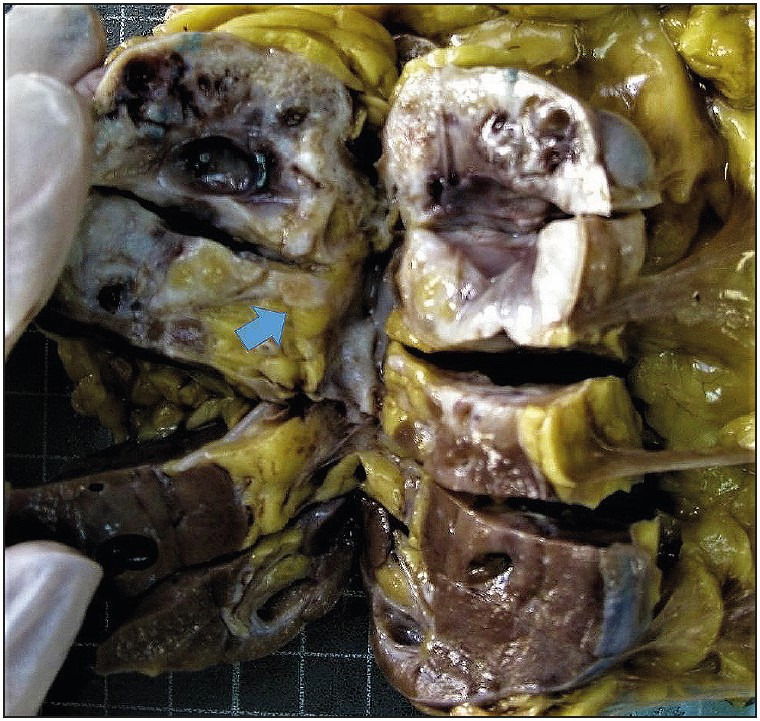
Macroscopic image of a MESTF case, which has solid component predominance. The tumor border is distinct and partly irregular, the tumor is bulging into renal sinus (arrow).

The mean tumor diameter was 6.7 (3.5-19) cm. The tumors were localized in the cortico-medullary, cortical and medullary regions in 7 (63.6%), 3 (27.3%) and 1 (9.1%) cases, respectively. In 7 (63.6%) cases, tumors appeared as a multicystic mass consisting of multiple simple cysts of varying size, uniloculated, containing serous fluid; solid component between them was very scarce. In one case (9.1%), both components were almost equal to each other. In 3 (27.3) cases including the male patient, tumors were usually cream-white in color, hard consistency, lobulated and solid in appearance, and contained few cystic structures up to 1.5 cm in diameter. The tumors were well circumscribed in 8 (72.7%) cases. Tumor borders were distinct but irregular in 3 (27.3%) cases and 2 of the tumors were bulging into renal sinus (Table 2) (Figure 1).

**Table 2:** Macroscopic and general histopathological features of the MESTF cases.

	**Case 1**	**Case 2**	**Case 3**	**Case 4**	**Case 5**	**Case 6**	**Case 7**	**Case 8**	**Case 9**	**Case 10**	**Case 11**
**Tumor size**	4 cm	6 cm	4 cm	5.5 cm	19 cm	4.3 cm	3.5 cm	4.2 cm	16 cm	4 cm	3.5 cm
**Tumor localization**	medullar	cortico-medullar	cortico-medullar	cortical	cortico-medullar	cortical, exophytic	cortical, exophytic	cortico-medullar	cotico-medullar	cortico-medullar	cortico-medullar
**Tumor borders**	distinct but irregular	distinct but irregular	well circumscribed	well circumscribed	well circumscribed	well circumscribed	well circumscribed	well circumscribed	well circumscribed	distinct but irregular	well circumscribed
**Capsule**	-	-	partial	complete	partial	-	complete	complete	partial	-	partial
**Capsule content**	-	-	SM	fibrous, SM	SM	-	fibrous, SM	fibrous, SM	fibrous	-	fibrous
**Dominant feature**	multicystic	multicystic	multicystic	multicystic	cystic-solid	multicystic	solid	solid	multicystic	solid	multicystic
**Epithelial component (%)**	90	65-70	80-85	90	45-50	85-90	30	10	85	20-25	70
**Stromal component (%)**	10	30-35	15-20	10	50-55	10-15	70	90	15	75-80	30
**Atypia**	focally mild epithelial	focally mild epithelial and stromal	focally mild epithelial and stromal	focally mild epithelial and stromal	focally mild epithelial and stromal	focally mild epithelial and stromal	focally mild epithelial	focally mild epithelial and stromal	focally mild epithelial	focally mild epithelial and stromal	focally mild epithelial and stromal
**Extra-parenchymal spread**	tumor bulging into renal sinus but no invasion	absent	absent	absent	absent	absent	absent	absent	absent	tumor bulging into renal sinus but no invasion	absent

**SM:** Smooth muscle.

### Histopathological Features

**Figure 2:**
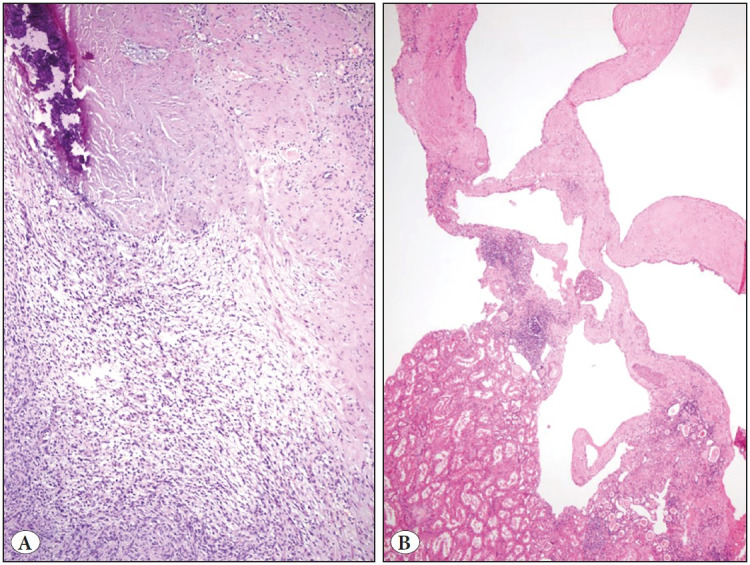
Tumor borders. **A)** Capsule with variable thickness, containing smooth muscle and dystrophic calcification around the cellular stromal tumor component (HE x100), **B)** Cystic tumor component separated from the kidney parenchyma by an irregular border (HE x40).

Seven cases had a partial or complete capsule of variable thickness (Figure 2). Five of them contained smooth muscle. The epithelial component was dominant in 7 cases (63.6%), the stromal component was dominant in 3 cases (27.3%); both components were almost equal in one case (9.1%). Mild epithelial atypia in the form of scattered foci was observed in all cases and most of them (72.7%) also had similarly stromal atypia (Table 2). Necrosis and notable mitotic activity were not observed in any case.

**Figure 3:**
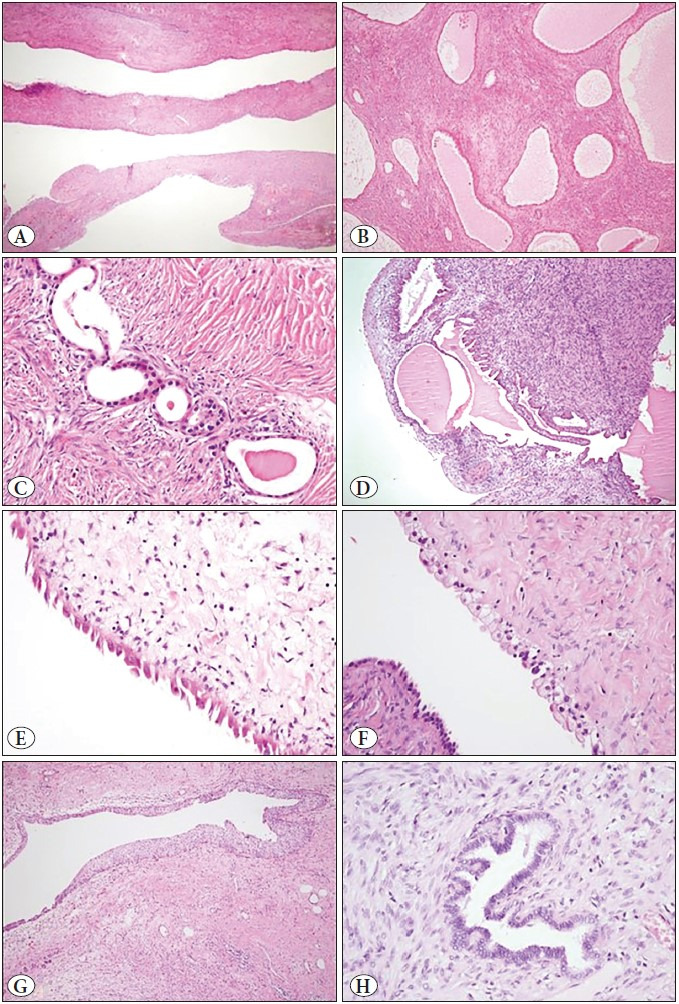
Features of epithelial component **A)** Flat type epithelium lining the collapsed macrocysts (HE x40), **B)** Microcysts surrounded by ovarian-type stroma (HE x100), **C)** Tubular structures lined by cuboidal epithelium (HE x400), **D)** Complex glandular structure with cystic papillary appearance (HE x100), **E)** Hobnail type epithelium lining the cyst (HE x400), **F)** Epithelium with foamy cytoplasm lining part of the cyst lumen (HE x400), **G)** Urothelial metaplasia of the lining epithelium (HE x100), **H)** Glandular structure lined with epithelium that has müllerian features (in tubal appearance) (HE x400).

In the epithelial component, the macrocyst was the dominant pattern in all cases. Varying proportions of microcysts and glandular structures accompanied. Rare tubulopapillary pattern and short cell cords (collapsed tubules) were seen in 4 and 3 cases, respectively. Flat and cuboidal type lining epithelium was observed in all cases; hobnail type epithelium was also observed in 10 of them. Columnar epithelium was only seen in 2 cases. The cell cytoplasm was usually amphophilic and eosinophilic. In some cases, a small amount of foamy and/or clear cytoplasm was also seen. Focal urothelial metaplasia of the lining epithelium was observed in 3 cases. In one case, several glandular structures lined with epithelium that had müllerian features (in tubal appearance) were observed. Epithelial component features were summarized in Table 3 and demonstrated in Figure 3.

**Table 3:** Epithelial component features of MESTF cases.

	**Case 1**	**Case 2**	**Case 3**	**Case 4**	**Case 5**	**Case 6**	**Case 7**	**Case 8**	**Case 9**	**Case 10**	**Case 11**
**Common pattern**	macrocyst > microcyst**≈** tubular *	macrocyst > microcyst≈ tubular	macrocyst > microcyst≈ tubular	macrocyst > microcyst≈ tubular	macrocyst > microcyst≈ tubular	macrocyst > microcyst≈ tubular	macrocyst > microcyst≈ tubular	macrocyst > microcyst≈ tubular	macrocyst > microcyst≈ tubular	macrocyst > microcyst≈ tubular	macrocyst > microcyst≈ tubular
**Complex structure**	-	-	-	rare TP**	rare TP	-	rare TP	rare TP	-	-	-
**Short cell cords (collapsed tubules)**	-	present	present	-	-	-	-	-	-	present	-
**Common epithelium type**	flat > cuboidal	flat > cuboidal > hobnail	flat ≈ cuboidal ≈ hobnail	flat ≈ cuboidal≈ hobnail	flat ≈ cuboidal ≈ hobnail	flat ≈ cuboidal ≈ hobnail	flat > cuboidal > hobnail	flat ≈ cuboidal > hobnail	flat ≈ cuboidal> hobnail	flat ≈ cuboidal > hobnail	cuboidal > hobnail > flat
**Columnar epithelium**	-	-	-	-	few	-	few	-	-	-	-
**Common cytoplasmic feature**	eosinophilic > amphophilic	amphophilic > eosinophilic	amphophilic ≈ eosinophilic	amphophilic ≈ eosinophilic	amphophilic ≈ eosinophilic	amphophilic ≈ eosinophilic	amphophilic > eosinophilic	amphophilic ≈ eosinophilic	amphophilic ≈ eosinophilic	amphophilic ≈ eosinophilic	amphophilic > eosinophilic
**Foamy cytoplasm**	-	few	few	few	-	notable	-	-	notable	-	-
**Clear cytoplasm**	-	few	few	-	-	few	-	-	notable	-	-
**Urothelial metaplasia**	-	-	-	-	notable	-	notable	-	-	notable	-
**Müllerian metaplasia**	-	-	-	-	-	-	notable	-	-	-	-

* “macrocyst > microcyst≈ tubular” means the most common pattern is macrocyst, microcyst and tubular patterns are equivalent to each other but less than macrocyst. This formula is valid for the whole table. **TP: Tubulopapillary pattern.

**Figure 4:**
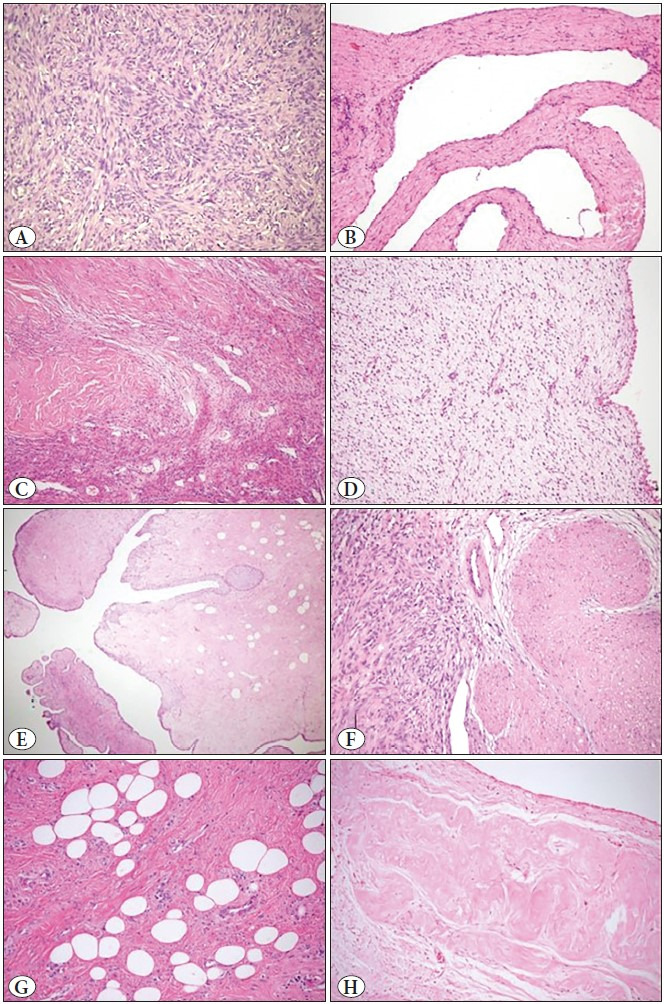
Features of stromal component **A)** Ovarian-type cellular stroma (HE x200), **B)** Collagenous stroma with septal characteristics (HE x200), **C)** Variable stroma with collagenous and cellular areas (HE x100), **D)** Hypocellular stroma with myxoid change (HE x200), **E)** Phyllodes-type architecture observed focally (HE x40), **F)** Smooth muscle metaplasia in nodular form (HE x200), **G)** Lipomatous metaplasia (HE x200), **H)** Corpus albicans-like nodular hyalinization (HE x200).

In 7 cases, the stromal component was variable and included hypocellular and cellular areas. In all of them, hypocellular areas were predominant and in the form of fibrous stroma with extensive collagenization, and 3 of them had also myxoid change. The cellular areas were mostly concentrated around the epithelial component and usually had an appearance of ovarian-type stroma. In 2 cases, the stromal component was almost entirely hypocellular and consisted mostly of collagenous and minimally ovarian-type stroma. In the other 2 cases, the stromal component was uniform and cellular; it consisted mostly of ovarian-type stroma in one case and spindle cells arranged in fascicles in the other, like spindle cell tumor*. *Pericystic, and/or corpus albicans-like nodular or patchy hyalinization was observed in 8 cases. Focal phyllodes-type architecture, smooth muscle metaplasia, dystrophic calcification, lipomatous and osseous metaplasia were other features observed. In addition, accompanying thick-walled vessels and various inflammatory reactions were seen in most of the cases. Stromal component features were summarized in Table 4 and demonstrated in Figure 4.

**Table 4:** Stromal component features of MESTF cases.

	**Case 1**	**Case 2**	**Case 3**	**Case 4**	**Case 5**	**Case 6**	**Case 7**	**Case 8**	**Case 9**	**Case 10**	**Case 11**
**Cellularity**	almost hypocellular	hypocellular > cellular *	hypocellular > cellular	hypocellular > cellular	hypocellular > cellular	hypocellular > cellular	cellular	cellular	almost hypocellular	hypocellular > cellular	hypocellular > cellular
**Features of hypocellular areas**	collagenous	collagenous	collagenous	collagenous+ myxoid change	collagenous+ myxoid change	collagenous	-	-	collagenous	collagenous+ myxoid change	collagenous
**Features of cellular areas**	ovarian-type > SM-type	ovarian-type	ovarian-type > SM-type	ovarian- type	ovarian- type > SM-type	ovarian-type	ovarian-type> SM-type	spindle cell tumor-like	ovarian-type	spindle cell tumor-like, SM-type	spindle cell tumor-like, SM-type
**Localization of cellular areas**	focal minimal	around epithelial structures	around epithelial structures	around epithelial structures	diffuse	around epithelial structures	diffuse	diffuse	focal minimal	scattered foci	scattered foci
**Focal phylloides-type pattern**	-	-	present	-	present	-	present	-	-	-	-
**Hyalinization**	CA-like foci	pericystic	pericystic, CA-like foci	pericystic, CA-like foci	pericystic	pericystic, CA-like foci	-	-	pericystic, CA-like foci	scattered foci	-
**SM metaplasia**	present	-	present	-	present	-	present	-	-	present	present
**Lipomatous metaplasia**	-	-	-	-	present	-	-	-	-	present	-
**Osseous metaplasia**	-	-	-	-	-	-	present	-	-	present	-
**Thick-walled vessels**	present	present	present	-	present	-	present	-	-	present	present
**Dystrophic calcification**	present	present	-	present	-	present	present	-	present	present	-
**Inflammatory reaction**	focally, MNC	-	-	xanthomatous reaction	focally, MNC	-	diffuse, MNC	focally, MNC+ E	focally, mixed**	focally, MNC	diffuse, mixed

* “hypocellular > cellular” means hypocellular areas are more than cellular areas. This formula is valid for the whole table. **Mixed: mononuclear cells and neutrophils, **SM**: Smooth Muscle, **CA:** Corpus Albicans, **MNC:** Mononuclear cells, **E:** Eosinophil.

### Immunohistochemistry

The stromal component was positive for both ER and PR in 9 of the female cases**,** and the staining was diffuse in all but one case that had focal positivity. In the other female patient, PR was focal positive, but ER was negative.

**Figure 5:**
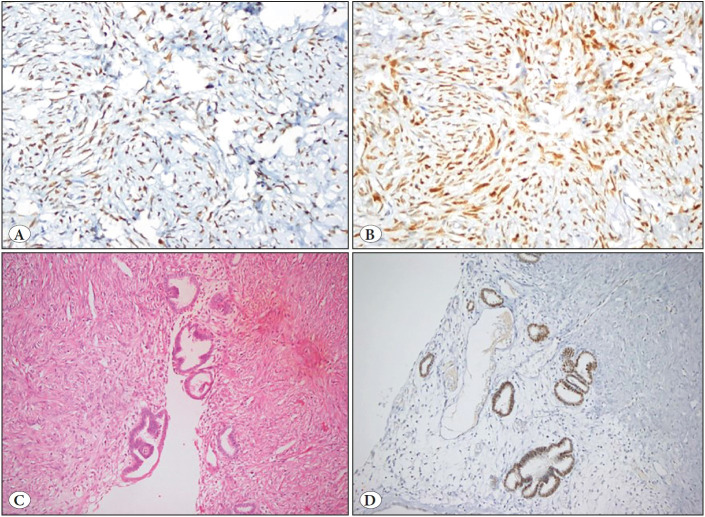
Immunohistochemical panel. **A,B)** The positivity for estrogen and progesterone receptor in the stromal component. **A:** ER x400, **B:** PR x400. **C,D)** Positivity for androgen receptor in the lining epithelium that has müllerian features. **C:** H&E x200, **D:** AR x200.

In the male patient*,* the stromal component was negative for ER and PR. Although both receptors were also negative in the epithelial component, in the examined preparation, focal positive staining for AR was observed in the lining epithelium that has mostly müllerian features (in tubal appearance). Immunohistochemical features were demonstrated in Figure 5.

## DISCUSSION

The clinicopathological experience about MESTF cases is limited, due to their rare nature. These tumors were mostly reported in middle-aged perimenopausal women (1-3). However, it can be seen in men (14–16). The female:male ratio has been reported as 2:1 for ACN (9). In a series of 53 MEST cases, this ratio was found to be 6.6:1, and the median age in males was shown to be higher than in females (49 in females and 71 in males) (1). Including this study, in small series and the case reports, the age range has been reported as 19-82 in men (2,3,14–16). Unlike the literature, 4 of our female cases (40%) were in the reproductive period, and the remaining cases were in the peri- or postmenopausal period. One of our cases was a 45-year-old man.

Although the histogenesis is not clearly known, it is thought that neoplastic transformation could have developed from müllerian remnants (4). The fact that it is common in women who have hormonal imbalance or received hormone therapy and in men with long-term sex-steroid exposure suggests that there may be a relationship between steroid hormones and MESTF cases (2,3,12). However, there are also studies in which this relationship cannot be demonstrated (17). A history of 2 years of hormone replacement therapy was determined in only one of our cases where clinical information was available.

While some of the cases occurred with nonspecific symptoms, some of them were detected incidentally (2,3,9). All of our cases presented with findings such as flank pain, abdominal pain, abdominal mass, hematuria and polyuria as reported in the literature.

MESTF cases are described as solitary, unilateral, well circumscribed, fluid-density masses with multiple septations on contrast-enhanced CT (9). Bilateral and multiple cases are rare (18). All of our cases were unilateral and interestingly most of them (72.7%) were located in the middle part of the kidney. Most of them (72.7%) were localized in the cortico-medullary or medullar region rather than the cortex, similar to previous reports (7). Our cases with known radiological features were mostly described as complicated cysts with regular borders.

Macroscopically, the tumor had an expansive border in 72.7% of our MESTF cases, while the others’ borders were irregular. All of them exhibited a combination of solid and cystic areas in variable proportions, as in the literature (1,3). It has been reported that some tumors may compress the pelvicalyceal system but true sinus fat infiltration is rare (9). Two of our tumors that had irregular borders were bulging into the renal sinus but did not have true fat invasion.

Both components show diversity in these tumors with biphasic characteristics. In this study, the epithelial component was dominant in 63.6% and the stromal component was dominant in 27.3% of the cases including the male case. The components were almost equal in one case. All histopathological features we found in our cases, which were reevaluated in detail, were in parallel with the literature (1,3,4).

For the epithelial component, macrocysts, microcysts and tubular pattern were frequent patterns. Tubulopapillary structures and short cell cords were rare patterns. Flat, cuboidal and hobnail epithelium were the most frequent but columnar epithelium was also observed in 2 (18.2%) of the cases. The epithelium-dominant areas with tubular and tubulopapillary pattern had more cuboidal-columnar cells, while the epithelium that lined the cysts was more flattened, as described in the literature (3). Hobnail cells that lined the cysts were also seen in varying proportions in almost all cases. Urothelial metaplasia was seen in 3 (27.3%) cases and müllerian metaplasia was seen in only one (9.1%) case. The cell cytoplasm was usually amphophilic and eosinophilic; however, a small number of clear or foamy cells were also observed in some cases.

The stromal component of MESTF cases is characterized by a spindle cell proliferation ranging from hypocellular to cellular areas (3). In one study, hypocellular stroma was found to be significantly more common in larger tumors and cellular stroma was found to be significantly more common in smaller tumors. It was thought that there was active proliferation in the stroma when the lesion was small, and fibrous stroma was more dominant as the size increased (1). In our series, the mean tumor size was 6.7 (3.5-19) cm and hypocellular stroma was dominant except in 2 cases. One of the cellular tumors had the smallest size in our series, and the other was also below average in size.

Smooth muscle stroma, which is a frequent stromal feature, was described in smaller tumors, but no statistical relationship has been demonstrated (1). In our study, 6 (54.5%) of the tumors had smooth muscle metaplasia in the stroma and these were in variable amounts.

Lipomatous metaplasia has been reported to be significantly related with larger tumors (1). There were 2 (18.2%) cases with lipomatous metaplasia and one of them was the largest tumor (19 cm) in our series.

The 8 (72.7%) cases of ours showed hyalinization as pericystic and/or corpus albicans-like nodular or rarely scattered foci. In addition, accompanying dystrophic calcification, thick-walled vessels, and various inflammatory reactions were seen in most of the cases. Focal phyllodes-type architecture and osseous metaplasia were other rare features that we observed.

In MESTs, the ER and PR positivity in the stromal component was reported as 73% and 85%, respectively (1). In another study, these rates were 62% and 85% in MESTs, while 19% and 40% in ACNs (4). In our study, stromal PR positivity was observed in all female cases. ER positivity was seen in 9 (90%) of female cases. In the male case, the stromal component was negative for both receptors.

We observed focal positive staining for AR in the lining epithelium, with mostly müllerian features, in the male case. Maclean et al. showed that AR was expressed in the epithelial cells of the fallopian tube regardless of menopausal status and cyclic phase in premenopausal women (19). In another study, Kamal et al. showed that postmenopausal endometrial epithelial cells had significantly higher AR expression compared to proliferative endometrium (20). As supported by the literature data, we considered AR positivity in the epithelium, which showed mostly müllerian features, as an ordinary finding in our case.

Multilocular cystic renal neoplasia with low malignant potential, cystic renal cell carcinoma, tubulocystic carcinoma, and angiomyolipoma with epithelial cysts should be evaluated in the differential diagnosis of MESTF cases with a complex cystic mass, and definitive diagnosis can be made only with pathological evaluation (9). Adult nephroblastoma, mesoblastic nephroma, sarcomatoid renal cell carcinoma, and metanephric adenofibroma should be considered in the differential diagnosis in more solid tumors (3,11). Some infectious etiologies such as renal abscess, aspergillosis and echinococcus can be excluded clinically (9).

MESTs are generally benign and surgical resection is sufficient for treatment (1,12). Malignant MEST cases have been reported rarely, and malignancy may have epithelial or stromal components. Malignant transformation of the stromal component consists of synovial sarcoma, rhabdomyosarcoma, chondrosarcoma, and unclassified sarcoma (14). Malignancies of the epithelial component consist of undifferentiated large cell carcinoma, mucinous borderline tumor and endometrioid adenocarcinoma (11). Carcinosarcoma arising in MEST has also been reported (21). Although the information on this subject is limited, it has been reported that aggressive malignant MESTF cases may respond to chemotherapy (13).

In this study, although mild atypia in the form of scattered foci in both epithelial and stromal components was frequently observed, no features indicating malignancy such as necrosis, increased mitotic activity, and diffuse and severe atypia were found in any of the cases.

After partial or radical nephrectomy, 10 of our 11 patients, who could be followed up for 4-258 months, were alive and healthy.

In conclusion, MESTF, which has distinctive features, should be in consideration during the differential diagnosis of cystic kidney tumors.

## Conflict of Interest

There is no conflict of interest.
